# Identifying optimal candidates for autologous peripheral blood stem cell therapy in patients with decompensated liver cirrhosis: a prognostic scoring system

**DOI:** 10.1186/s13287-023-03622-y

**Published:** 2024-01-02

**Authors:** Siyuan Tian, Guanya Guo, Xia Zhou, Yansheng Liu, Gui Jia, Linhua Zheng, Lina Cui, Kemei Wang, Miao Zhang, Keshuai Sun, Shuoyi Ma, Chunmei Yang, Xinmin Zhou, Changcun Guo, Yulong Shang, Ying Han

**Affiliations:** 1 State Key Laboratory of Cancer Biology, National Clinical Research Center for Digestive Diseases, Xijing Hospital of Digestive Diseases, Air Force Military Medical University, The Fourth Military Medical University, Xi’an, 710032 Shaanxi China; 2 Department of Gastroenterology, The Air Force Hospital From Eastern Theater of PLA, Nanjing, 210002 Jiangsu China

**Keywords:** Stem cell therapy, Liver cirrhosis, Nomogram, Risk stratification

## Abstract

**Background:**

Stem cell transplantation shows great potential to improve the long-term survival of cirrhosis patients. However, therapeutic effects may not be homogeneous across the whole study population. This study constructed an easy-to-use nomogram to improve prognostic prediction and aid in treatment decision making for cirrhotic patients.

**Methods:**

From August 2005 to April 2019, 315 patients with decompensated cirrhosis receiving autologous peripheral blood stem cell (PBSC) transplantation were enrolled in this study. They were randomly classified into training (2/3) and validation (1/3) groups. A predictive model was developed using Cox proportional hazard models and subsequently validated. The predictive performance of the model was evaluated and also compared with other prognostic models.

**Results:**

Age, creatinine, neutrophil-to-lymphocyte ratio, and Child–Turcotte–Pugh class were included in the nomogram as prognostic variables. The nomogram showed high discrimination power concerning the area under receiver operating characteristic curves (3/5-year AUC: 0.742/0.698) and good consistency suggested by calibration plots. Patients could be accurately stratified into poor- and good-outcome groups regarding liver-transplantation free survival after receiving PBSC therapy (*P* < 0.001). Compared with poor-outcome group, the liver function of patients listed for liver transplantation in the good-outcome group was significantly improved (*P* < 0.001). Besides, our nomogram achieved a higher C-index (0.685, 95% CI 0.633–0.738) and better clinical utility compared with other conventional prognostic models.

**Conclusions:**

The proposed nomogram facilitated an accurate prognostic prediction for patients with decompensated cirrhosis receiving PBSC transplantation. Moreover, it also held the promise to stratify patients in clinical trials or practice to implement optimal treatment regimens for individuals.

**Supplementary Information:**

The online version contains supplementary material available at 10.1186/s13287-023-03622-y.

## Background

According to the Global Burden of Disease Report, nearly 1.32 million fatalities were attributed to liver cirrhosis in 2017, representing 2.4% of global all-cause mortality [1]. Moreover, the prevalence of decompensated cirrhosis presents a persistently increasing trend during the past 30 years, and the 5-year mortality rate due to severe complications of advanced cirrhosis can be as high as 18–80% [2]. At present, liver transplantation remains the only definitive treatment for patients with decompensated cirrhosis [3]. However, only a minority of the patients could receive transplantation due to donor shortage, and the clinical outcome is still not satisfactory [4].

New possibilities for treating cirrhosis have emerged due to recent advancements in stem cell biology [5, 6]. Stem cells are characterized by the capacity of self-renewal, immunomodulation, and multipotent differentiation [7]. In 2005, stem cell transplantation was first utilized for liver disease, which provided a novel therapeutic approach in the clinical setting [8]. Stem cell-based therapies have since advanced rapidly and attracted significant interest in the field of hepatology, including liver cirrhosis. Among them, peripheral blood stem cell (PBSC) transplantation stands out as a feasible option because of its low risk and high tolerability for patients [9]. Most of the previous studies have confirmed that stem cell therapy could improve liver biochemical parameters [10–13]. However, some researchers drew a contrary conclusion, like the study conducted by Newsome et al. [14]. On the one hand, the small sample size and lack of assessment of long-term clinical outcomes prevent definitive conclusions regarding the effect of this treatment on cirrhosis. On the other hand, the heterogeneities of the patients varying in etiology, course of the disease as well as immune status should also be taken into full consideration, which may give rise to these varied clinical observations and treatment outcomes of individual patients. In contrast, our previous studies with up to 10 years of follow-up confirmed that patients with decompensated cirrhosis had an elevated chance of survival after PBSC transplantation [15]. However, there is no individual prognostic tool to provide survival estimates for patients with cirrhosis after stem cell therapy to guide individualized therapeutic strategies.

So, we conducted this retrospective cohort study to specifically identify the prognostic factors of patients with decompensated cirrhosis receiving PBSC transplantation, by analyzing demographic data, etiology of liver disease, as well as clinical features. By integrating these variables, we developed and validated a predictive tool, which could facilitate prognostic prediction and inform therapy choice in clinical application.

## Methods

### Study design and population

From August 2005 to April 2019, we recruited 333 patients with decompensated cirrhosis, who completed autologous PBSC transplantation in the Xijing Hospital. Specifically, the criteria for selection were as follows: (1) age range from 18 to 75 years; (2) decompensated cirrhosis (hepatitis B or C); (3) Child–Turcotte–Pugh (CTP) score of 7 or greater; (4) antiviral therapy for a minimum of 6 months prior to PBSC transplantation. Patients were excluded based on the following: (1) previous diagnosis within the last 30 days of spontaneous peritonitis, hepatic encephalopathy, or variceal hemorrhage; (2) hepatorenal syndrome; (3) hepatocellular carcinoma or other malignancies; (4) alcohol abuse. Clinical symptoms, upper digestive endoscopy, ultrasonography, or computerized tomography were used to diagnose decompensated cirrhosis [16]. Chronic hepatitis B or C was diagnosed based on patient history, serologic tests, and biochemical indicators. Patients with unknown survival information were excluded from the analysis. All of the eligible patients were randomly assigned a number by a computer program, and thus they were classified into the training or the validation group at a 2:1 ratio. Informed consent was obtained from all patients and the study was approved by the Ethics Committee of Xijing Hospital.

### Treatment procedures

The procedure of PBSC transplantation was described previously [15]. In brief, G-CSF was administered to the patients subcutaneously once per day at a dosage ranging from 5 to 10 mg/kg/d for a total of 4 consecutive days. Leukapheresis with the COBER Spectra Apheresi System (Gambro BCT, Inc, Stockholm, Sweden) was employed to harvest the mobilized PBSCs. Then, patients received 50 mL of leukapheresis products comprising 10^7^–10^8^ cells per kg of body weight administered into their hepatic arteries. Following leukapheresis, the cell preparations were examined by flow cytometry to assess the percentages of CD14^+^ and CD34^+^ cells. CD14^+^ cells accounted for around 90% of the newly extracted PBMCs, whereas CD34^+^ cells accounted for 20% of the total cells. There were at least 50 ml of newly extracted PBMCs comprising 6 × 10^8^ cells. In each procedure, over 1 × 10^8^ CD34^+^ cells were transplanted into the recipient's liver via the hepatic artery. Every participant received only one PBSC treatment. The procedure protocol was standardized and strictly followed. All patients received standard medications like antiviral treatment, and complications such as hepatoencephalopathy, hepatorenal syndromes, variceal hemorrhage, and ascites were managed in compliance with updated guidelines on the basis of PBSC transplantation [16–18].

### Data collection and outcomes

The electronic medical records were retrieved for all pertinent clinical and demographic information, such as age, sex, etiology of liver disease, blood routine test, liver, and kidney function test, as well as coagulation parameters. As described previously [15], biochemical indexes were set as dichotomous variables, with the reference being the normal range of healthy people. The neutrophil-to-lymphocyte ratio (NLR) was defined as the absolute neutrophil count divided by the absolute lymphocyte count. The X-tile software (version 3.6.1) was used to identify the optimal cutoff values for NLR, which were then transformed into categorical variables. The CTP [19], ALBI [20], and model for end-stage liver disease (MELD) [21] score were calculated according to the relevant formulas. Baseline data were collected during patients’ first visit and they were regularly followed up by outpatient visits or telephone calls. The end point of each patient was recorded as liver transplantation (LT) or death. Patients who remained alive without LT at last follow-up date (January 1st, 2020) or lost to follow up were censored. LT-free survival was defined as the duration between the initiation of PBSC treatment and liver transplantation or death/censure.

### Statistical analysis

SPSS 23.0 software (SPSS, IBM) and R software version 4.1.1 were applied in analyzing statistical data. With the use of X-tile software (version 3.6.1), we derived the optimal cutoff value for NLR and risk score. Median with interquartile range (IQR) and/or mean ± (SD) were employed to present continuous variables. As deemed appropriate, we contrasted continuous variables by means of the Student’s t test or the Mann–Whitney U test. Quantitative comparisons of categorical variables, which were expressed as numbers with percentages, were made using the χ2 test or Fisher's exact test. For paired data that did not follow a normal distribution, a Wilcoxon-matched pair test was applied. To determine independent prognostic factors linked to LT-free survival, the Cox proportional hazards model was used. In the subsequent multivariate analysis, we took into account the factors that were significant in the previous univariate analysis. The predictive model was finally determined by the backward and forward stepwise approach based on the Akaike information criterion (AIC). A Kaplan–Meier (KM) curve was generated, and the log-rank test was utilized to determine the differences (variations) in survival rates. Then, using the “rms” package included in R software, a nomogram was developed as per the results of a multivariate analysis carried out on the training dataset. Measurements of discrimination and calibration were taken from the training dataset as well as the validation dataset to further analyze the performance of the nomogram. The time-dependent ROC curves were plotted with the “timeROC” R package, and 3- and 5-year AUC values of the ROC curve were calculated. In addition, the calibration curve was employed for visualization, and within the curve, the 45-degree line was found to provide the most accurate prediction. Moreover, the discriminatory ability of the nomogram was compared with three conventional prognostic models for liver disease (CTP, ALBI, and MELD score) by calculating C-index. The clinical significance of these models was evaluated with the help of decision curves by quantifying the net benefits at different probability thresholds. A *P*-value (two-sided) of < 0.05 denoted the significance threshold.

## Results

### Baseline characteristics

During the study period, a total of 333 patients met the inclusion and exclusion criteria, 18 patients were excluded due to incomplete survival information. Finally, 315 eligible patients (training cohort: *N*1 = 210, validation cohort: *N*2 = 105) were included in the analysis (Additional file 1: Fig. S1). The patterns of other missing values are illustrated in Additional file 1: Fig. S2. The baseline characteristics of all patients are summarized in Table 1. The median follow-up was 50.5 (IQR 13.6–86.5) months and 47.6 (IQR 17.3–81.5) months in the training and validation cohorts, correspondingly. Patients were predominantly male (69.8%) with a median age of 48 (IQR 42–56) years. The hepatitis B virus accounted for the majority of the etiology of liver cirrhosis. No significant variation was found between the training cohort and validation cohorts in terms of demographic data and other clinicopathological parameters except that patients in the training cohort recorded a slightly elevated level of serum bilirubin. The LT-free survival of patients was displayed in Additional file 1: Fig. S3, and no significant variation existed across the groups. In the entire cohort, the median value of NLR was 1.53 (IQR 1.02–2.48). The optimal cutoff value of NLR is 2.7, as determined by X-tile software. Compared with patients with NLR > 2.7, patients with NLR ≤ 2.7 had a more favorable prognosis (Additional file 1: Fig. S4).

**Table 1:** Demographic data and baseline characteristics of the study cohort

Baseline characteristics	Entire cohort	Training cohort	Validation cohort	*P*-value
	(*N* = 315)	(*N*1 = 210)	(*N*2 = 105)	
Gender, *n* (%)				0.224
Female	95 (30.2)	68 (32.4)	27 (25.7)	
Male	220 (69.8)	142 (67.6)	78 (74.3)	
Age (years)	48 (42–56)	48 (42–56)	47 (41–56)	0.512
Etiology, *n* (%)				0.669
HBV	288 (91.4)	191 (91.0)	97 (92.4)	
HCV	27 (8.6)	19 (9.0)	8 (7.6)	
PLT, 10^9^/L				0.895
≥ 100	43 (13.7)	29 (13.8)	14 (13.3)	
< 100	271 (86.0)	180 (85.7)	91 (86.7)	
ALT, IU/L, *n* (%)				0.375
≤ 40	181 (57.5)	117 (55.7)	64 (61.0)	
> 40	134 (42.5)	93 (44.3)	41 (39.0)	
AST, IU/L, *n* (%)				0.130
≤ 54	161 (51.1)	101 (48.1)	60 (57.1)	
> 54	154 (48.9)	109 (51.9)	45 (42.9)	
Albumin, g/L, *n* (%)				0.804
≥ 30	114 (36.2)	75 (35.7)	39 (37.1)	
< 30	201 (63.8)	135 (64.3)	66 (62.9)	
TBIL, μmol/L, *n* (%)				0.032
≤ 17.1	52 (16.5)	28 (13.3)	24 (22.9)	
> 17.1	263 (83.5)	182 (86.7)	81 (77.1)	
Creatinine, μmol/L, *n* (%)				0.755
≤ 106	293 (93.0)	196 (93.3)	97 (92.4)	
> 106	22 (7.0)	14 (6.7)	8 (7.6)	
Sodium, mmol/L, *n* (%)				0.946
≥ 137	242 (76.8)	160 (76.2)	82 (78.1)	
< 137	67 (21.3)	44 (21.0)	23 (21.9)	
PT (s)				0.444
≤ 15	23 (7.3)	17 (8.1)	6 (5.7)	
> 15	292 (92.7)	193 (91.9)	99 (94.3)	
Fibrinogen, g/L, *n* (%)				0.875
≥ 2	232 (73.7)	155 (73.8)	77 (73.3)	
< 2	82 (26.0)	54 (25.7)	28 (26.7)	
INR				0.293
≤ 1.5	131 (41.6)	83 (39.5)	48 (45.7)	
> 1.5	184 (58.4)	127 (60.5)	57 (54.3)	
NLR	1.53 (1.02–2.48)	1.50 (1.01–2.44)	1.61 (1.06–2.68)	0.233
Ascites, *n* (%)				0.832
None	44 (14.0)	31 (14.8)	13 (12.4)	
Mild	123 (39.0)	82 (39.0)	41 (39.0)	
Moderate to massive	148 (47.0)	97 (46.2)	51 (48.6)	
CTP class, *n* (%)				0.686
B	185 (58.7)	125 (59.5)	60 (57.1)	
C	130 (41.3)	85 (40.5)	45 (42.9)	
CTP score	9.0 (8.0–11.0)	9 (8.0–11.0)	9 (8.0–11.0)	0.955
MELD score	14.2 (11.4–17.2)	14.2 (11.9–17.0)	14.3 (10.5–17.7)	0.561
ALBI score	− 1.42 ± 0.40	− 1.43 ± 0.38	− 1.43 ± 0.44	0.160
Survival status, *n* (%)				0.294
Alive	182 (57.8)	117 (55.7)	65 (61.9)	
Dead/liver transplantation	129 (41.0)/4 (1.2)	90 (42.9)/3 (1.4)	39 (37.0)/1 (1.0)	
Follow-up duration, months	49.5 (15.8–86.0)	50.5 (13.6–86.5)	47.6 (17.3–81.5)	0.982

*HBV* hepatitis B virus, *PLT* platelet, *ALT* alanine aminotransferase, *AST* aspartate aminotransferase, *TBIL* total bilirubin, *PT* prothrombin time, *INR* international normalized ratio, *NLR* neutrophil-to-lymphocyte ratio, *CTP* Child–Turcotte–Pugh, *MELD* model for end-stage liver disease, *ALBI* albumin–bilirubin

### Prognostic factors for LT-free survival

Univariate and multivariate regression analysis were conducted in the training cohort to identify prognostic factors for patients receiving PBSCs transplantation. The results of univariate analysis revealed that age, creatinine, NLR, CTP class, and MELD score were significantly associated with the prognosis of patients. Subsequently, these variables were imported into a multivariate stepwise Cox regression analysis to ascertain the extent of their independent influence on clinical outcomes (Table 2). The AIC was computed to evaluate the relative goodness of fit and the simplicity of the model before making the final selection. Finally, we picked the dominant model containing all four variables, since it had the lowest AIC value (Additional file 1: Table S1). Furthermore, survival curves stratified by these factors are shown in Additional file 1: Fig. S5, demonstrating significant difference in survival.

**Table 2:** Univariate and multivariate Cox regression analysis of prognostic factors for liver-transplantation free survival in the training cohort

Variable	Univariate cox regression		Multivariate cox regression	
	HR (95% CI) *P*-value		HR (95% CI) *P*-value	
Gender				
Female	Reference			
Male	1.222 (0.788–1.897)	0.371	–	–
Age (years)				
≤ 50	Reference		Reference	
> 50	1.838 (1.221–2.767)	0.004	1.826 (1.207–2.761)	0.004
Etiology of cirrhosis				
HBV	Reference			
HCV	0.805 (0.389–1.666)	0.560	–	–
PLT, 10^9^/L				
≥ 100	Reference			
< 100	1.338 (0.672–2.663)	0.407	–	–
ALT, IU/L				
≤ 40	Reference			
> 40	0.871 (0.578–1.312)	0.509	–	–
AST, IU/L				
≤ 54	Reference			
> 54	1.122 (0.745–1.690)	0.581	–	–
Albumin, g/L				
≥ 30	Reference			
< 30	1.017 (0.667–1.552)	0.937	–	–
TBIL, μmol/L				
≤ 17.1	Reference			
> 17.1	1.102 (0.612–1.985)	0.747	–	–
Creatinine, μmol/L				
≤ 106	Reference		Reference	
> 106	2.750 (1.316–5.748)	0.007	2.906 (1.375–6.141)	0.005
Sodium, mmol/L				
≥ 137	Reference			
< 137	0.954 (0.574–1.586)	0.857	–	–
PT (s)				
≤ 15	Reference			
> 15	1.037 (0.453–2.374)	0.932	–	–
Fibrinogen, g/L				
≥ 2	Reference			
< 2	0.956 (0.597–1.531)	0.851	–	–
INR				
≤ 1.5	Reference			
> 1.5	0.973 (0.643–1.472)	0.895	–	–
NLR				
≤ 2.7	Reference		Reference	
> 2.7	3.490 (2.245–5.427)	< 0.001	3.086 (1.973–4.828)	< 0.001
Ascites, *n* (%)				
None	Reference			
Mild	1.064 (0.560–2.022)	0.850	–	–
Moderate to massive	1.197 (0.648–2.212)	0.567	–	–
CTP class				
B	Reference		Reference	
C	1.722 (1.144–2.591)	0.009	1.734 (1.145–2.626)	0.009
MELD score				
< 15	Reference			
≥ 15	1.615 (1.075–2.429)	0.021	–	–
ALBI grade				
2	Reference			
3	0.973 (0.646–1.464)	0.895	–	–

*HBV* hepatitis B virus, *PLT* platelet, *ALT* alanine aminotransferase, *AST* aspartate aminotransferase, *TBIL* total bilirubin, *PT* prothrombin time, *INR* international normalized ratio, *NLR* neutrophil-to-lymphocyte ratio, *CTP* Child–Turcotte–Pugh, *MELD* model for end-stage liver disease, *ALBI* albumin–bilirubin, *HR* hazard ratio, *CI* confidence interval

### Nomogram construction and validation

**Fig. 1:**
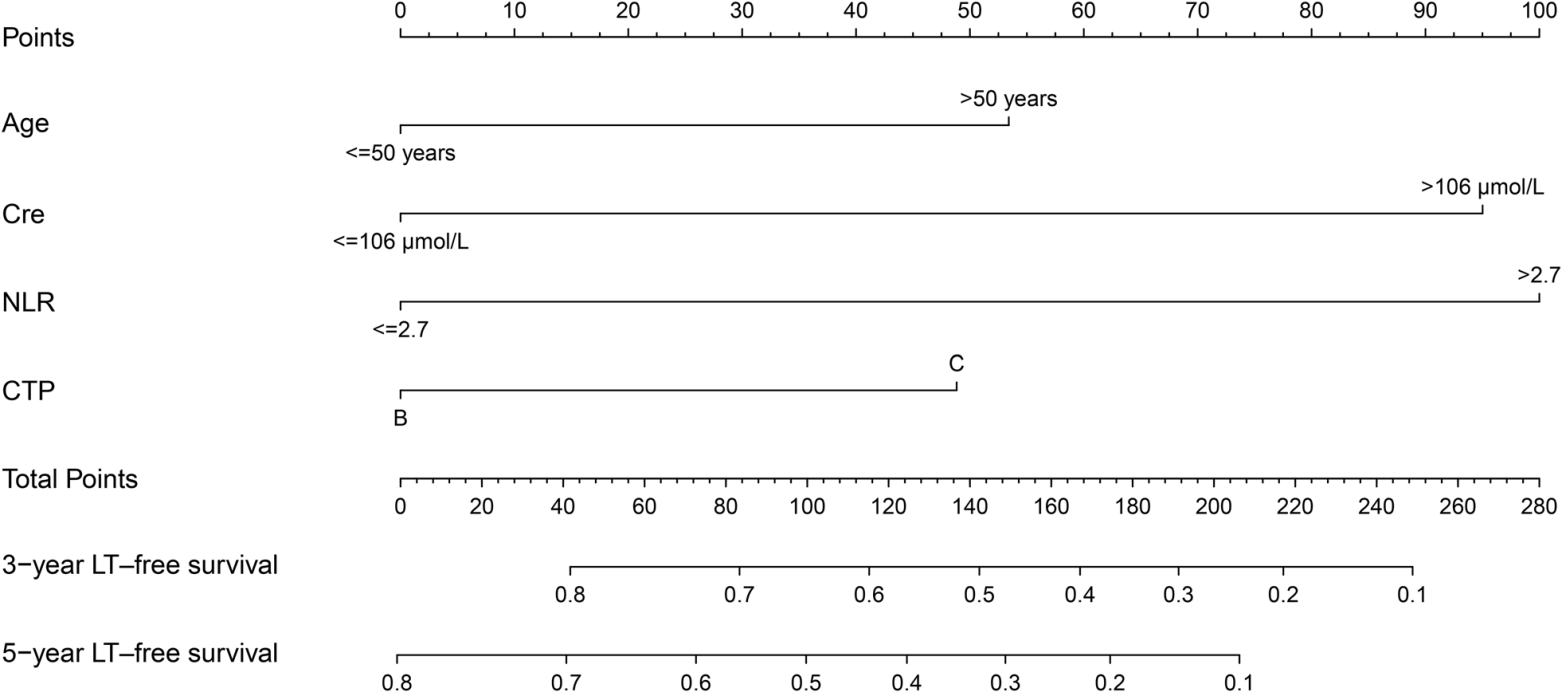
Nomogram for predicting the 3- and 5-year LT-free survival of cirrhosis patients receiving PBSC transplantation. *Cre* Creatinine, *NLR* neutrophil-to-lymphocyte ratio, *CTP* Child–Turcotte–Pugh, *LT-free survival* Liver-transplantation free survival, *PBSC* peripheral blood stem cell

**Fig. 2:**
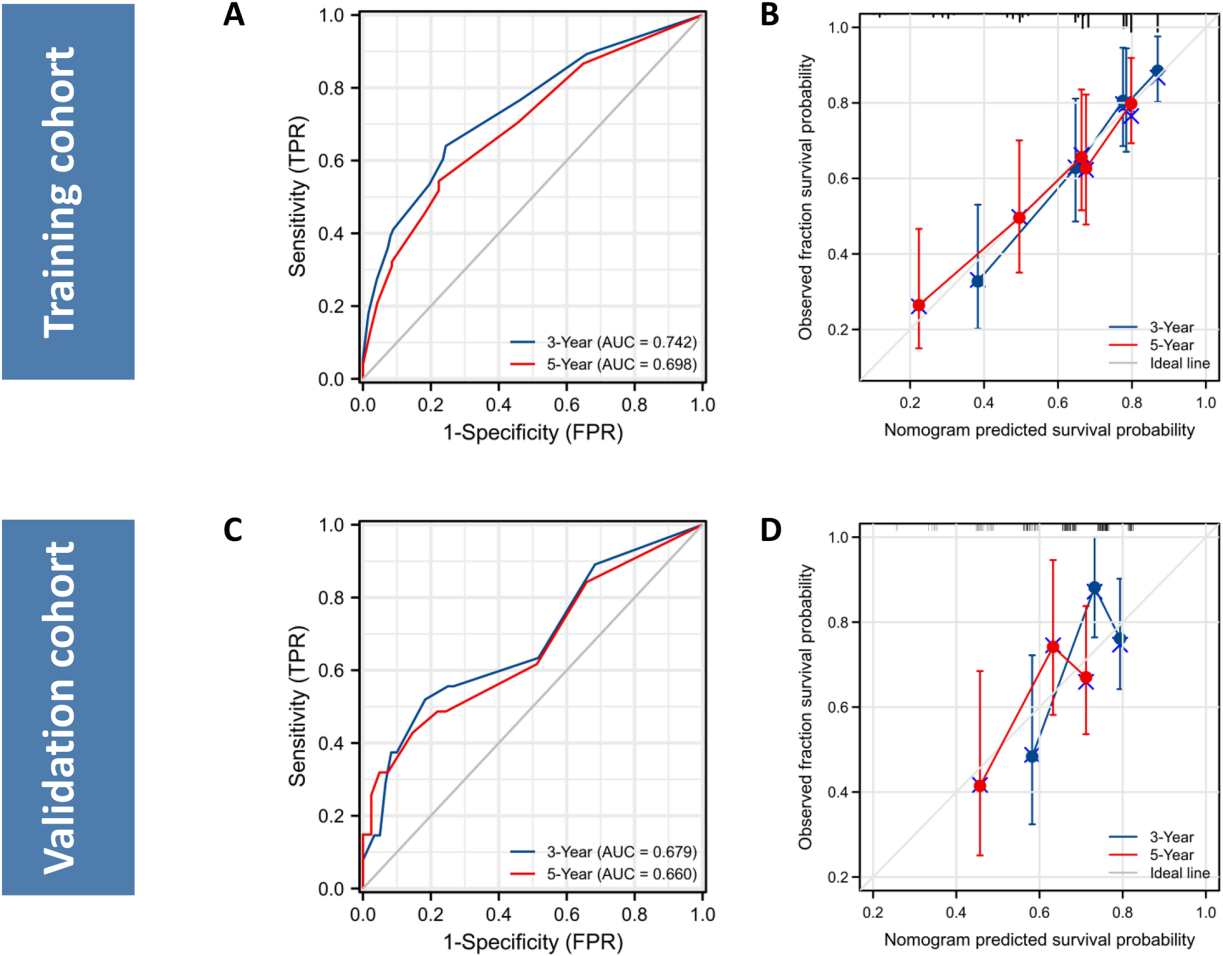
Evaluation of the predictive performance of the nomogram. **A** and **C** ROC curves for the prediction of 3- and 5-year LT-free survival, **B** and **D** Calibration curves for prediction of 3- and 5-year LT-free survival in the training and validation cohort. *AUC* area under the curve, *ROC* receiver operator characteristic, *LT-free survival* Liver-transplantation free survival

On the basis of these independent prognostic parameters, a nomogram characterized by scale line and scores weight reflected 3- and 5-year LT-free survival prediction was established (Fig. 1). The score corresponding to each nomogram variable was generated by the weight of each clinical factor in the multivariate regression analysis. The detail score for each variable is listed in Additional file 1: Table S2. We employed ROC curves and calibration curves to evaluate the performance of the nomogram. In the training cohort, the area under the ROC curve for predicting 3- and 5-year LT-free survival of patients was 0.742 and 0.698, respectively (Fig. 2A). Meanwhile, the accuracy of the nomogram remained satisfactory at the time points mentioned above in the validation cohort (Fig. 2C). Moreover, the calibration curves also demonstrated good probability consistencies between the predicted and observed values (Fig. 2B, D).

### Risk stratification and subgroup analysis

**Fig. 3:**
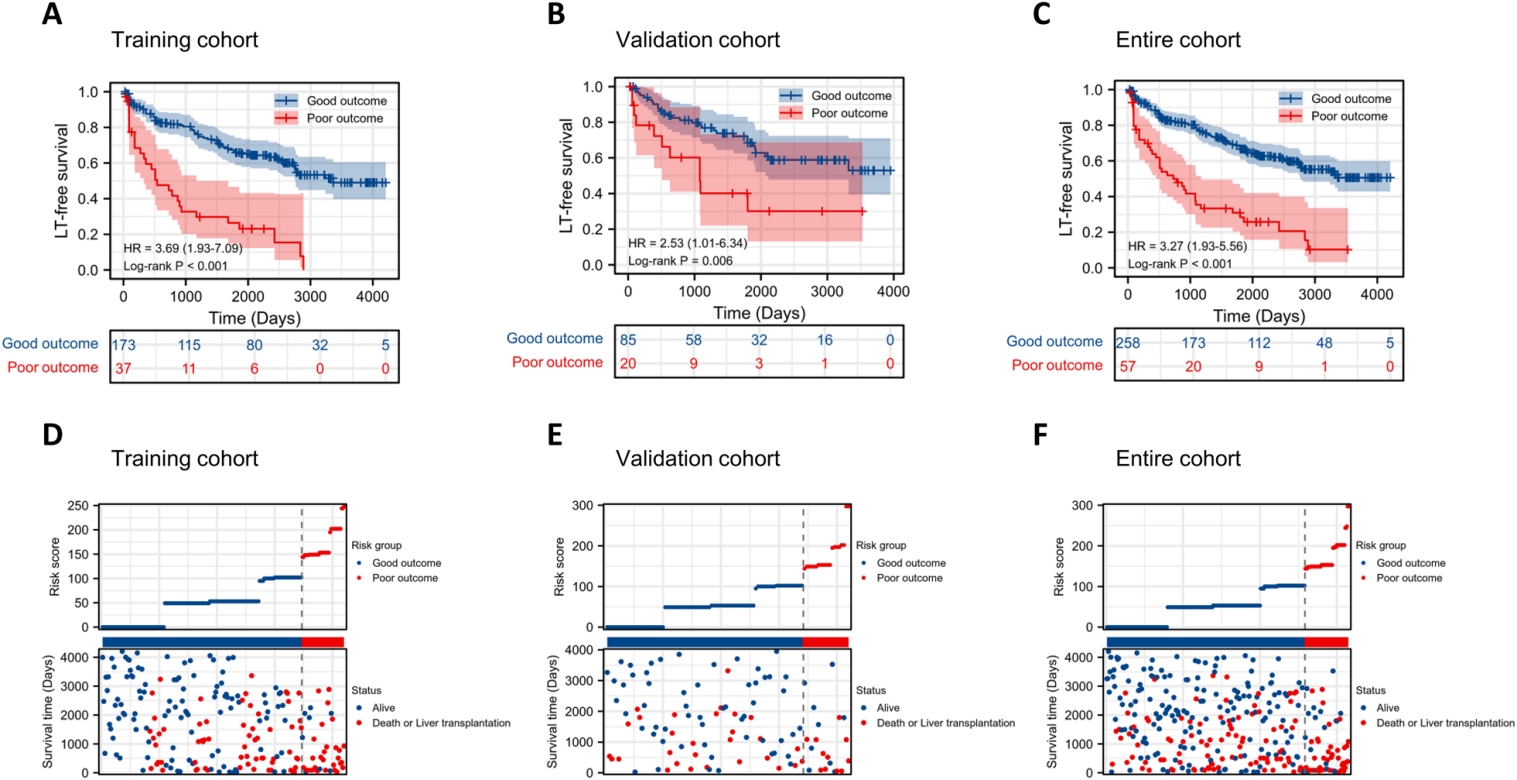
Survival analysis of LT-free survival in different risk subgroups stratified by the nomogram score. Kaplan–Meier curves of stratified survival in the **A** training, **B** validation and **C** entire cohorts. The distribution of risk score calculated by the nomogram and survival status of patients in the **D** training, **E** validation and **F** entire cohorts. Good outcome (nomogram score < 144) and poor outcome (nomogram score ≥ 144). LT-free survival, liver-transplantation free survival

By calculating the risk score, patients from the training cohort were stratified into two groups according to the optimal cutoff values determined by the X-tile software (Additional file 1: Fig. S6). Consequently, patients were divided into good-outcome group (score < 144) and poor-outcome group (score ≥ 144). To further verify the effectiveness of our model, we compared the survival difference within two risk subgroups in the training, validation and entire cohort. Additionally, Fig. 3A–C depicts that the survival curves were well separated, which indicated good discrimination in different subgroups. The survival status and risk score distribution are displayed in Fig. 3D–F. To reduce the possible risk of bias due to incomplete outcome data, we also performed a subgroup analysis with a complete follow-up of patients. Similarly, a significant difference in survival curves was also observed (Additional file 1: Fig. S7).

**Fig. 4:**
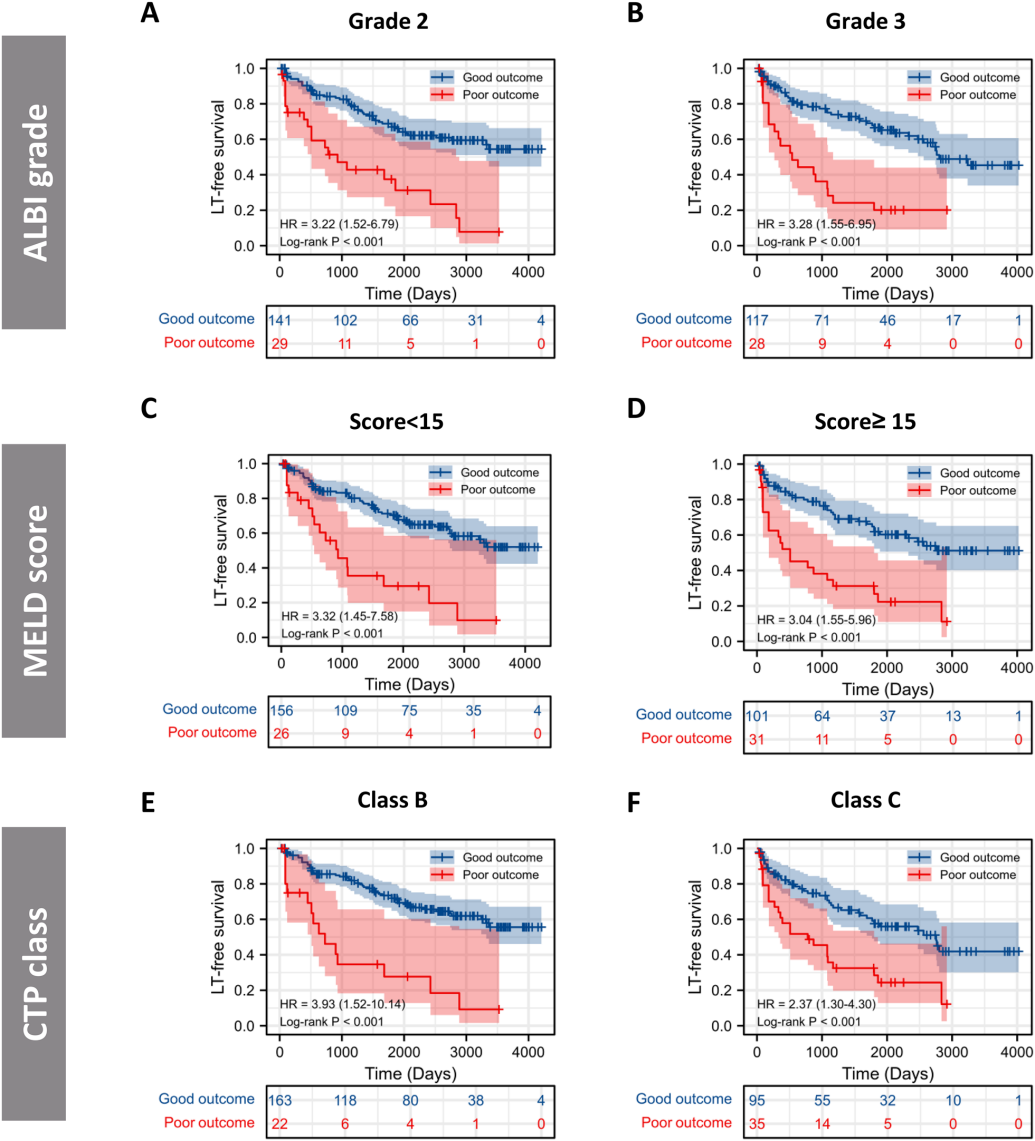
Subgroup analysis of LT-free survival in patients stratified by **A** ALBI grade, **B** MELD score and **C** CTP class. *CTP* Child–Turcotte–Pugh; *MELD* model for end-stage liver disease, *ALBI* albumin–bilirubin, *LT-free survival* Liver-transplantation free survival

**Fig. 5:**
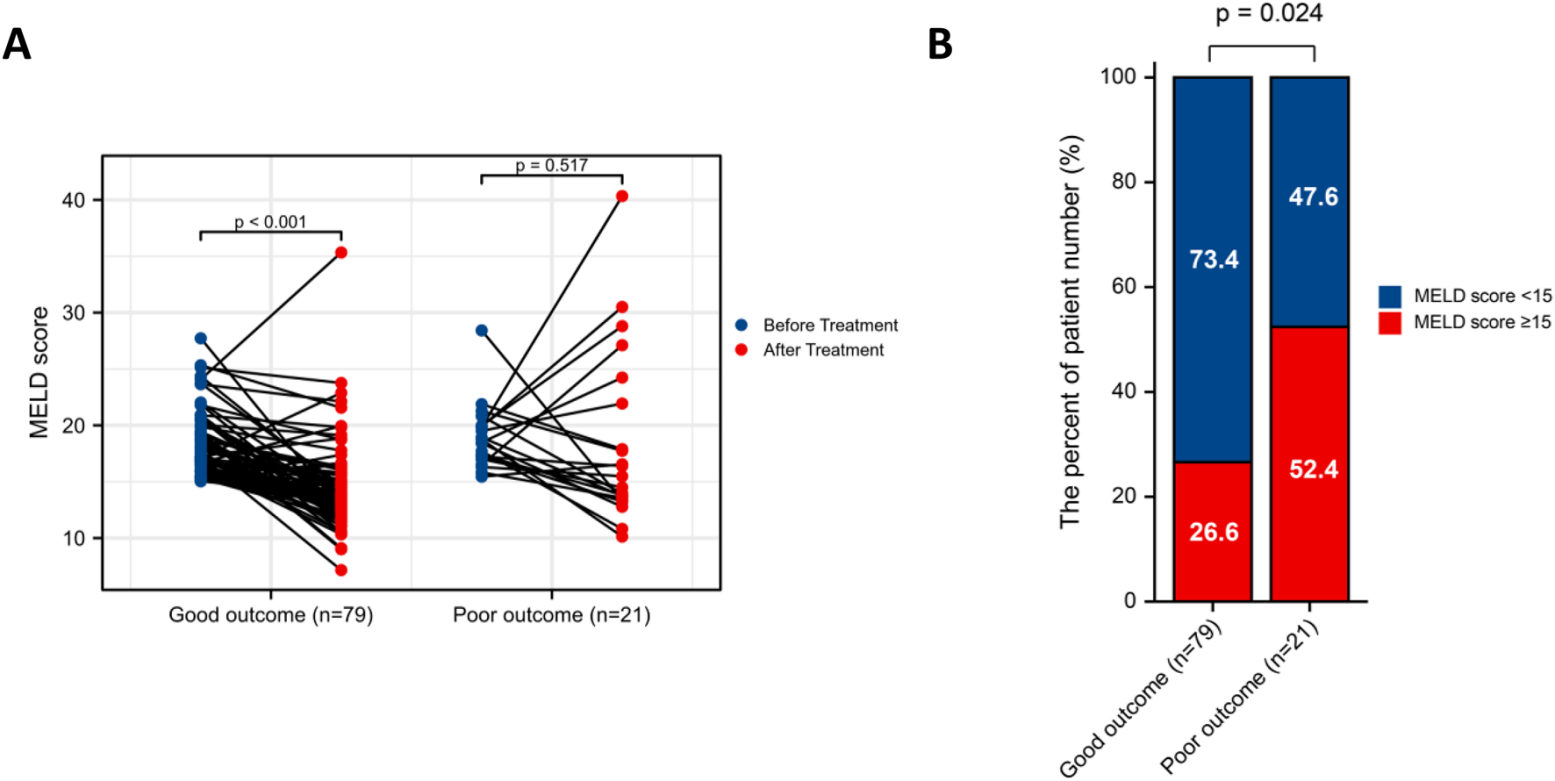
Influence of PBSC transplantation on the MELD score in patients listed for LT (MELD score > 15). **A** Improvement of MELD scores before and after the treatment in two groups. **B** The percentage-staked bar plots for the distribution of MELD score in two groups after PBSC therapy. *MELD* model for end-stage liver disease, *PBSC* peripheral blood stem cell, *LT* liver transplantation

To better assess the prognostic ability of the risk score, we performed a stratification analysis to confirm whether it retains its ability to predict LT-free survival in various subgroups. The good-outcome group showed better LT-free survival in both HBV and HCV subgroups in contrast with the poor-outcome group (Additional file 1: Fig. S8A–B). We also verified that the risk score retained its capability to distinguish patients with different clinical outcomes in either HBV-DNA (+) or HBV-DNA (−) patients (Additional file 1: Fig. S8C–D). Moreover, similar results were also obtained in subgroups analysis stratified by liver function classes including ALBI grade, MELD score as well as CTP class (Fig. 4A–F). Notably, among patients with baseline MELD score ≥ 15, significant improvements in MELD score were observed in the good-outcome group. However, no significant variation was observed in the poor-outcome group (Fig. 5A). In comparison, good-outcome group showed a higher proportion of MELD score < 15 following PBSC transplantation. The median follow-up time in good- and poor-outcome was 90 (IQR 46–237) and 68 (IQR 40–208) days, respectively. No significant variation was noted between the groups at the follow-up (*P* = 0.282).

### Comparison of the predictive performance and net clinical benefits with other models

The performance of the nomogram and other models including ALBI grade, MELD score, and CTP class were compared by calculating C-index or plotting DCA curves. The C-index value for the entire cohort of the nomogram was 0.685 (0.633–0.738), which was considerably superior in contrast with the other three models (Additional file 1: Table S3). Furthermore, to evaluate the nomogram's clinical significance, we conducted a decision curve analysis to quantify the net benefits. Compared with other models, the nomogram exhibited a satisfactory benefit at different threshold probabilities in predicting 3- and 5-year LT-free survival (Additional file 1: Fig. S9).

### Development of an online calculator for the prognostic evaluation

For user convenience, we also developed a web-based online calculator to assist clinicians in making survival estimates for those patients receiving PBSCs treatment. The online tool is available at: https://xijing-PBSC-model.shinyapps.io/DynNomapp/.

By selecting the corresponding clinical parameters, the predicted survival probability for the whole time period could be obtained. The screenshot of the web-based nomogram for the prediction of LT-free survival is shown in Additional file 1: Fig. S10.

## Discussion

In recent years, results from clinical trials and animal models show that stem cell therapy holds exciting therapeutic promise for hepatic fibrosis [22, 23]. Our previous study also demonstrated that autologous PBSC transplantation could improve the liver functions of patients with cirrhosis [11]. On the basis, we further evaluated the long-term effects of PBSC therapy. The results showed that PBSC transplantation is a safe treatment regimen for patients with cirrhosis and can improve long-term survival without increasing the risk of hepatocellular carcinoma [15]. A recent randomized controlled trial (RCT) also reported that mesenchymal stem cell therapy substantially enhanced the liver function and long-term survival rate of patients with HBV-associated decompensated liver cirrhosis [24]. However, existing research has not definitively shown the optimal cirrhosis population that would gain the most benefit from stem cell transplantation. In the current investigation, we developed and validated a prognostic model by integrating the superiorities of a large sample cohort with a follow-up duration of more than 10 years, which could accurately predict individual outcomes with favorable performance and discrimination. This scoring system was proposed to stratify patients into good- and poor-outcome groups with significantly different treatment outcomes. It will be helpful to guide clinicians to select the appropriate treatment option and control the heterogeneity of enrolled patients in future clinical trials.

In this study, age older than 50 years, serum creatinine > 106 μmol/L, NLR > 2.7, and CTP class C independently functioned as factors linked to the dismal survival of patients after PBSC transplantation. These factors are all easily accessible in the clinical practice. Currently, no related study has compared the therapeutic efficacy of stem cell transplantation on cirrhosis caused by different etiologies. In our study, only viral hepatitis-related cirrhotic patients were enrolled. Considering China’s status as a region with a high HBV disease burden, gaining deeper knowledge about the efficacy of stem cell therapy in the treatment of HBV-induced cirrhosis is crucial in improving the prognosis of patients in China. Besides, the performance status and organ function are also important factors affecting the effectiveness of stem cell therapy. Patients with older age, impaired organ function, and infections will just receive a limited survival benefit from the treatment. Similarly, Zhang et al. reported that the therapeutic effects of stem cell therapy for liver cirrhosis varied partly by patient age. Assessing patient age is necessary prior to clinical use of stem cell therapy [25].

NLR is a simply calculated and routinely available marker reflecting systemic inflammation. In our study, the cutoff value of the NLR was set as 2.7. Patients with NLR > 2.7 exhibited poor long-term prognosis. It was reported that NLR was intimately linked to the prognosis of patients including those with compensated or decompensated cirrhosis [26]. In addition, this index could also serve as an independent indicator of mortality in patients with liver failure awaiting transplantation [27, 28]. Neutrophils and lymphocytes are key components of the immune system. Elevated neutrophil count reflects ongoing inflammation, whereas decreased lymphocyte count is associated with immunosuppression. In recent years, mounting evidence suggests that the effectiveness of stem cell transplantation is primarily dependent on the microenvironment in vivo during liver damage and may be the key factor in determining how well a therapy will work [29]. We therefore speculate the immunosuppressive condition may be one of the attributes that hinders the efficacy of stem cell therapy.

Based on the above factors, a stepwise model selection procedure based on AIC was applied to construct the final nomogram. The nomogram showed good discriminative and calibration ability in both the training and validation cohort. Meanwhile, this individualized risk stratification of nomogram could contribute to identify subgroups where it would be advantageous for using PBSC transplantation to improve survival. The predictive power and clinical utility of the nomogram were also compared with some commonly used prognostic models. Our results further confirmed that this model was superior to CTP class, MELD score, and ALBI grade. The target population is the core issue of prognostic models, since it determines the different treatment or care option. Although the clinical application of stem cells has formally expanded, the recommended candidates have not yet been clearly defined. The heterogeneity of the study population may lead to the inconsistent outcomes of stem cell treatment on cirrhosis. Newsome et al. conducted a randomized controlled study and found that stem cell infusion did not improve liver dysfunction or fibrosis [20]. Notably, the majority of the people who participated in the research were older individuals who had cirrhosis, and the causes of their condition were multiple, including primary biliary cholangitis, non-alcoholic fatty liver disease, and alcohol-related liver disease. Based on our risk stratification model, those patients are likely to be classified as a poor-outcome group if they also suffered from other risk factors. Besides, some scholars pointed out that the dose and viability of transfused stem cells were lower than other studies showing an improvement in liver function and disease severity [30]. So, the result of that trial failed to support the potential efficacy of stem cell therapy. This also suggests to us that not all patients are the best target population and the implementation of stem cell therapy should be scrutinized. The current model may provide risk stratification criteria to identify the optimal candidate of PBSC transplantation.

There is no denying that liver transplantation is still the most effective treatment for patients with end-stage liver disease. Nevertheless, most patients do not undergo liver transplantation due to lack of donors, high costs, and immune rejection. As per the available data, nearly 40% of those eligible for a liver transplant have to wait over 2 years, and the lack of livers available for transplantation is directly responsible for the deaths of 10–15% of patients [31, 32]. Moreover, liver transplantation is not feasible for all cirrhosis patients. Since 2002, MELD scoring system has been employed for donor liver allocation in the USA [33]. In particular, patients with MELD scores < 15 are not the ideal candidates for liver transplantation [34]. The application of PBSC therapy is expected to fill the gap of treatment. At this time, our risk stratification model could be a useful tool in clinical trials to identify the target populations that could benefit from PBSC transplantation. Then, we could enroll these patients in clinical stem cell studies to reduce their potential risk of disease progression due to long waiting periods for suitable donor livers. After assessing the risk of the persistent shortage of appropriate liver donors, the model might potentially be used to select the optimal patients for PBSC therapy when the MELD score is ≥ 15. As seen in our results, 75% of patients among those with a MELD score ≥ 15 could be classified into a good-outcome group. Meanwhile, a remarkable enhancement in the MELD score was noted in the good-outcome group, with 73.4% of treated patients delisted for liver transplantation.

To the best of knowledge, this is the first nomogram for predicting the long-term outcomes of patients with cirrhosis after PBSC transplantation. Other strengths of our study lie in its relatively large sample size and the long-term follow-up period. We used the LT-free survival as the study end point, allowing better estimates of therapeutic efficacy of PBSC therapy. Nevertheless, several limitations were also present in this study. First, it was a retrospective study suffering from the possible selection bias. The patients who were lost to follow-up early may be different from those with complete follow-up, leading to different therapeutic effects. Thus, we performed a subgroup analysis with complete follow-up of patients and validated the performance of nomogram. Second, although the nomogram demonstrated good predictive performance, external validation was not performed. Despite advances in stem cell therapy for cirrhosis, there are still many parameters that need to be standardized from a clinical perspective. To some extent, it is challenging to find a representative cohort with a large sample size. Thus, an internal validation method was applied in our study. We plan to collect data from prospective multicenter studies to further validate our findings. Finally, we used some easily obtained clinical features and laboratory indicators to construct this model, aiming at identifying the optimal population for PBSC therapy. However, some important parameters were not fully explored in this study. In a recently published review [35], Yang et al. placed the factors influencing the efficacy of stem cell therapy into two main categories, which were cells factors (the sources, cell preparation procedure, dosages, engraftment routes) and patients factors (liver function, inflammation, age, etc.). We could notice that there are many factors affecting the therapeutic effect. In the current study, on the basis of our previous research, we paid more attention to the influence of patient factors on the therapeutic effects. The cells factors remain to be considered in future extensions of this model. Furthermore, finding some valuable biomarkers from specimens of patients using transcriptomics, proteomics, and metabolomics are essential next steps in future research.

## Conclusions

In summary, based on the large-scale data derived from real-world medical records, we developed and verified a prognostic nomogram, showing good performance with individual prediction. More importantly, this risk stratification tool will be helpful to assess outcomes in clinical practice and enhance patient stratification in future clinical trials to target the optimal population.

### Supplementary Information

**Additional file 1**. Supplementary tables and figures.

## Data Availability

The datasets used and/or analyzed during the current study are available from the corresponding author on reasonable request.

## Abbreviations

PBSC: Peripheral blood stem cell
NLR: Neutrophil-to-lymphocyte ratio
AIC: Akaike information criterion
HBV: Hepatitis B virus
PLT: Platelet
ALT: Alanine aminotransferase
AST: Aspartate aminotransferase
TBIL: Total bilirubin
PT: Prothrombin time
INR: International normalized ratio
IQR: Interquartile range
CI: Confidence interval
LT-free survival: Liver-transplantation free survival
AUC: Area under the curve
ROC: Receiver operator characteristic
