# Supplementary material for: Identifying optimal candidates for autologous peripheral blood stem cell therapy in patients with decompensated liver cirrhosis: a prognostic scoring system
Source: Stem Cell Res Ther. 2024 Jan 2;15:8. doi: 10.1186/s13287-023-03622-y (PMC10763677; doi:10.1186/s13287-023-03622-y)
Supplement: Supplementary file 1 — Additional file 1. Supplementary tables and figures. [file 13287_2023_3622_MOESM1_ESM.docx]

**Identifying Optimal Candidates for Autologous Peripheral Blood Stem Cell Therapy in Patients with Decompensated Liver Cirrhosis: A Prognostic Scoring System**

Siyuan Tian^1, #^, Guanya Guo^1, #^, Xia Zhou^1, #^, Yansheng Liu^1^, Gui Jia^1^, Linhua Zheng^1^, Lina Cui^1^, Kemei Wang^1^, Miao Zhang^1^, Keshuai Sun^2^, Shuoyi Ma^1^, Chunmei Yang^1^, Xinmin Zhou^1^ , Changcun Guo^1,*^, Yulong Shang^1, *^ and Ying Han^1, *^

**Supplementary materials**

Content

Supplementary Table 1. The possible models selected by AIC criterion……………...3

Supplementary Table 2. Detailed scores of all variables in the nomogram…………...4

Supplementary Table 3. Comparison of the predictive accuracy of the current nomogram with other models.………………………………………………….……...5

Supplementary Figure 1. Flow chart of the study design……………………………...6

Supplementary Figure 2. Patterns for missing data in the entire cohort………………7

Supplementary Figure 3. Survival analysis of patients receiving PBSC transplantation in the three cohorts…………………………………………………………………….8

Supplementary Figure 4. Identification of the optimal cut-off value of NLR for patients in the training cohort using the X-tile software……………………………...9

Supplementary Figure 5. Survival analysis of patients in the training cohort stratified by the variables associated with LT-free survival in multivariate analysis…………..10

Supplementary Figure 6. Identification of the optimal cut-off values of total scores for the nomogram using the X-tile software…………………………………………….11

Supplementary Figure 7. Survival analysis of LT-free survival in patients with complete follow-up…………………………………………………………………..12

Supplementary Figure 8. Survival analysis was performed for each subgroups in the entire cohort………………………………………………………………….……….13

Supplementary Figure 9. Decision curve analysis of the 3- and 5-year LT-free survival in the entire cohort……………………………………………………………………14

Supplementary Figure 10. The screenshot of the web-based nomogram for the prediction of LT-free survival………………………………………………………...15

**Supplementary Table 1. The possible models selected by AIC criterion.**

| **Model** | **Variables** | **AIC** |
| --- | --- | --- |
| 1 | Age + Cre + NLR + CTP class + MELD score | 856.69 |
| 2 | Age + Cre + NLR + CTP class | 855.61 |
| 3 | Age + Cre + NLR + MELD score | 855.83 |
| 4 | Age + NLR + CTP class + MELD score | 859.75 |
| 5 | Cre + NLR + CTP class + MELD score | 862.82 |
| 6 | Age + Cre + CTP class + MELD score | 876.67 |
| 7 | Age + NLR + CTP class | 859.66 |
| 8 | Age + Cre + NLR | 860.25 |
| 9 | Cre + NLR + CTP class | 861.74 |
| 10 | Age + Cre + CTP class | 874.84 |

AIC, Akaike Information Criterion; Cre, Creatinine; NLR, neutrophil-to-lymphocyte ratio; CTP, Child-Turcotte-Pugh; MELD, Model for End Stage Liver Disease.

**Supplementary Table 2. Detailed scores of all variables in the nomogram.**

| **Variable** | **Categories** | **Nomogram score** |
| --- | --- | --- |
| Age (years) | ≤50 | 0 |
|  | >50 | 53 |
| Cre (μmol/L) | ≤106 | 0 |
|  | >106 | 95 |
| NLR | ≤2.7 | 0 |
|  | >2.7 | 100 |
| CTP class | B | 0 |
|  | C | 49 |

Cre, Creatinine; NLR, neutrophil-to-lymphocyte ratio, CTP, Child-Turcotte-Pugh.

**Supplementary Table 3. Comparison of the predictive accuracy of the current nomogram with other models.**

| **Model** | **Entire cohort (n=314)** | |
| --- | --- | --- |
|  | **C-index (95% CI)** | **P-value vs. Nomogram** |
| Nomogram | 0.685 (0.633-0.738) | — |
| Versus CTP | 0.594 (0.535-0.654) | 0.005 |
| Versus MELD | 0.559 (0.506-0.612) | <0.001 |
| Versus ALBI | 0.567 (0.513-0.621) | <0.001 |

CTP, Child-Turcotte-Pugh; MELD, Model for End Stage Liver Disease; ALBI, albumin-bilirubin


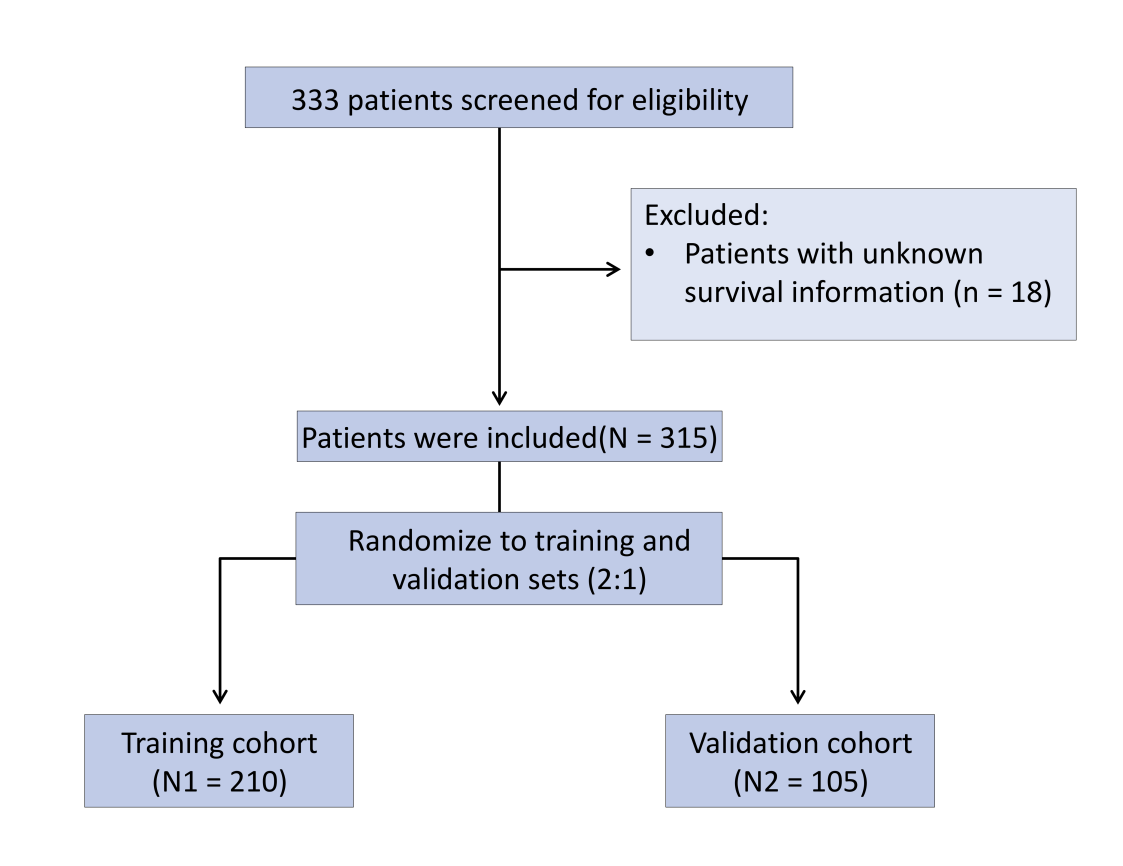


**Supplementary Figure 1. Flow chart of the study design.**

**
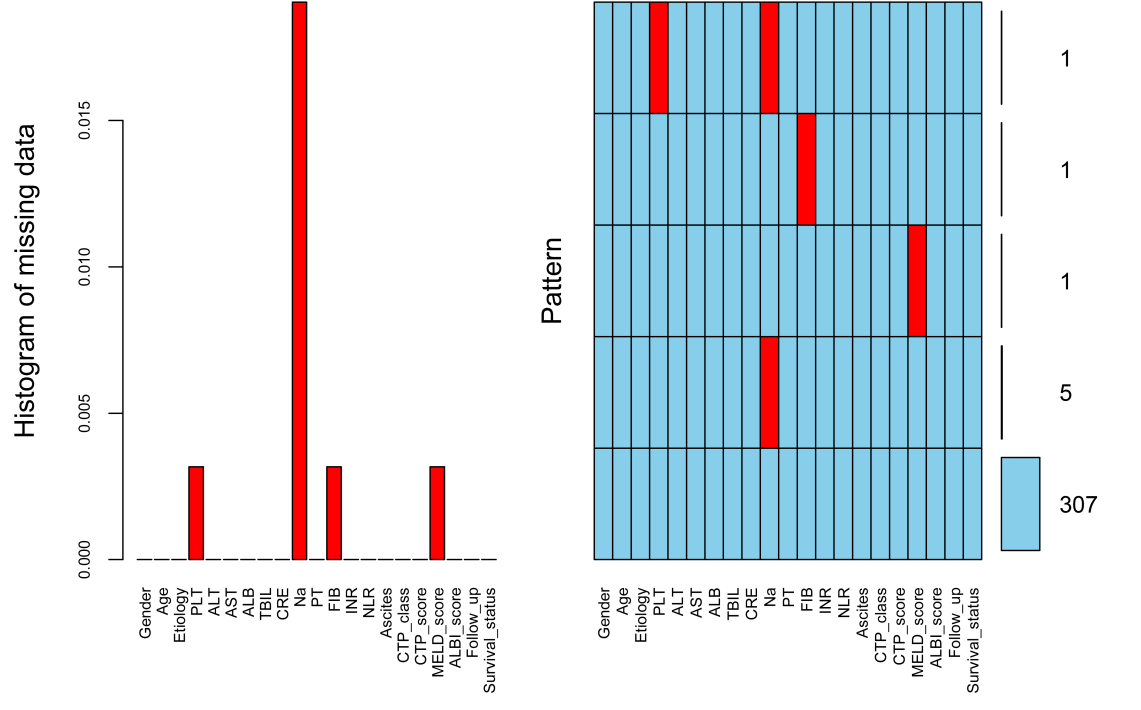
**

**Supplementary Figure 2. Patterns for missing data in the entire cohort.**

**
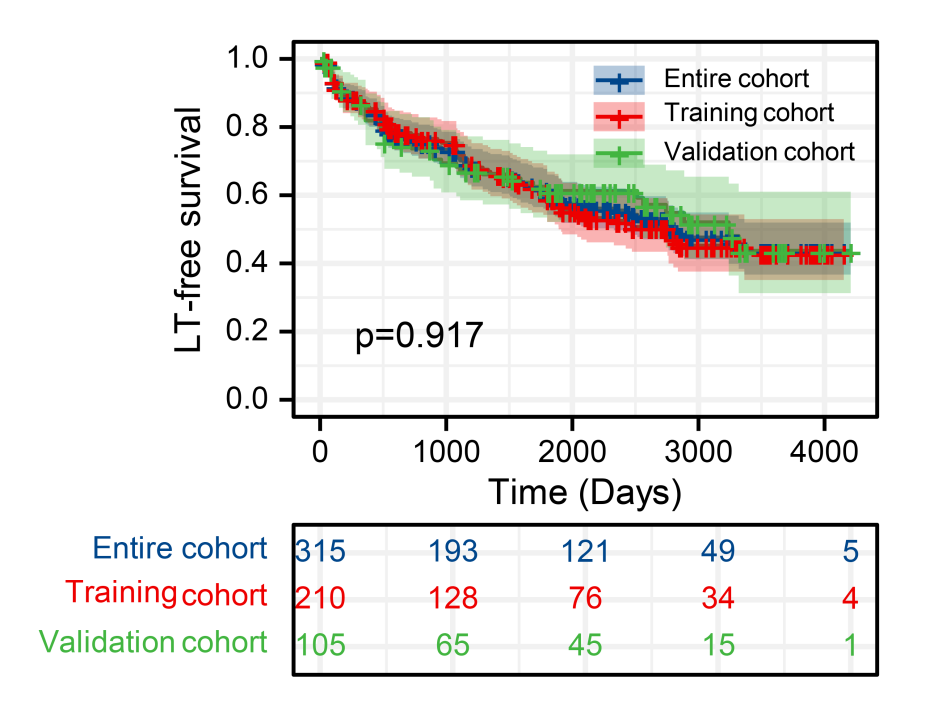
**

**Supplementary Figure 3. Survival analysis of patients receiving PBSC transplantation in the three cohorts.** PBSC, peripheral blood stem cell. LT-free survival, Liver-transplantation free survival.

**
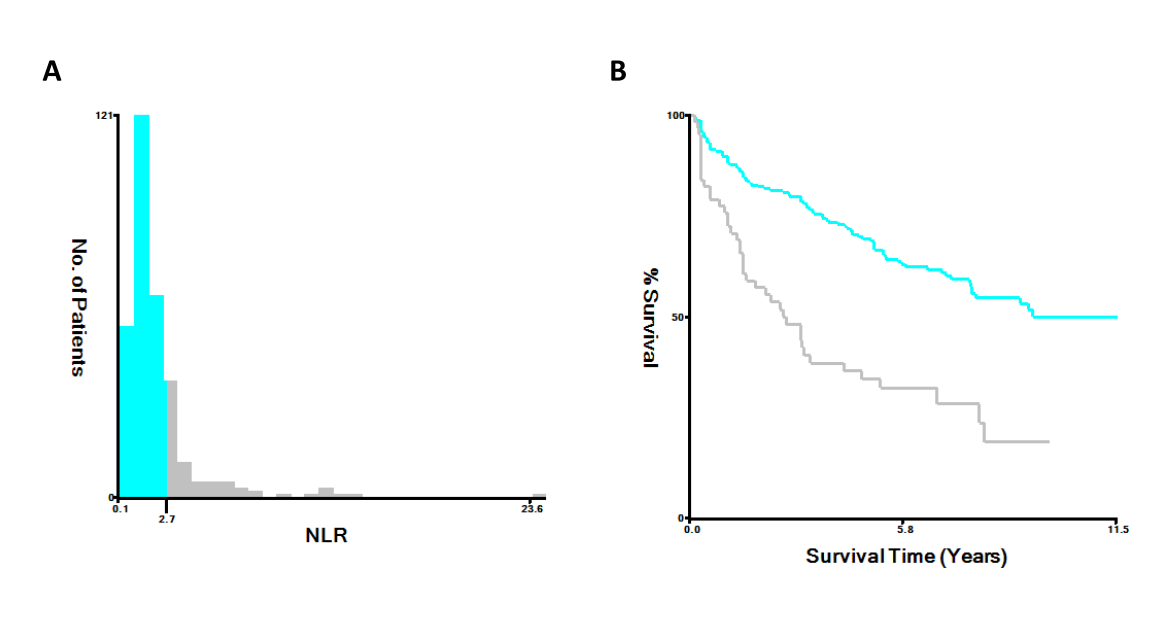
**

**Supplementary Figure 4. Identification of the optimal cut-off value of NLR for patients in the training cohort using the X-tile software.** (A) The histogram shows the optimal cut-off point of the NLR. (B) Kaplan-Meier curve corresponding to the cut-off point for the training cohort. NLR, neutrophil-to-lymphocyte ratio.

**
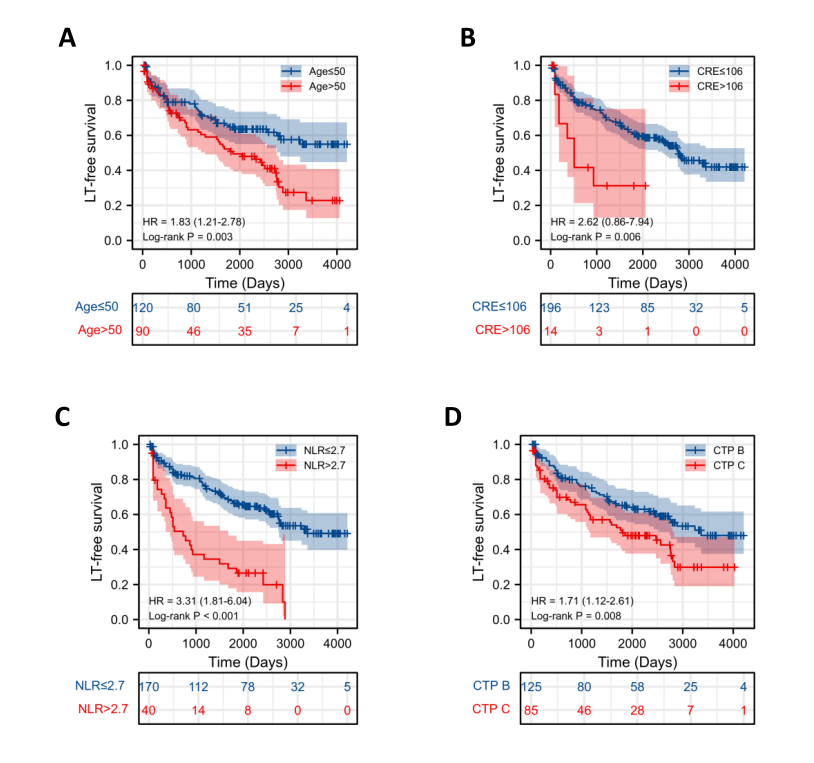
**

**Supplementary Figure 5. Survival analysis of patients in the training cohort stratified by the variables associated with LT-free survival in multivariate analysis.** (A) Age. (B) Cre. (C) NLR and (D) CTP class. Cre, Creatinine; NLR, neutrophil-to-lymphocyte ratio; CTP, Child-Turcotte-Pugh; LT-free survival, Liver-transplantation free survival.


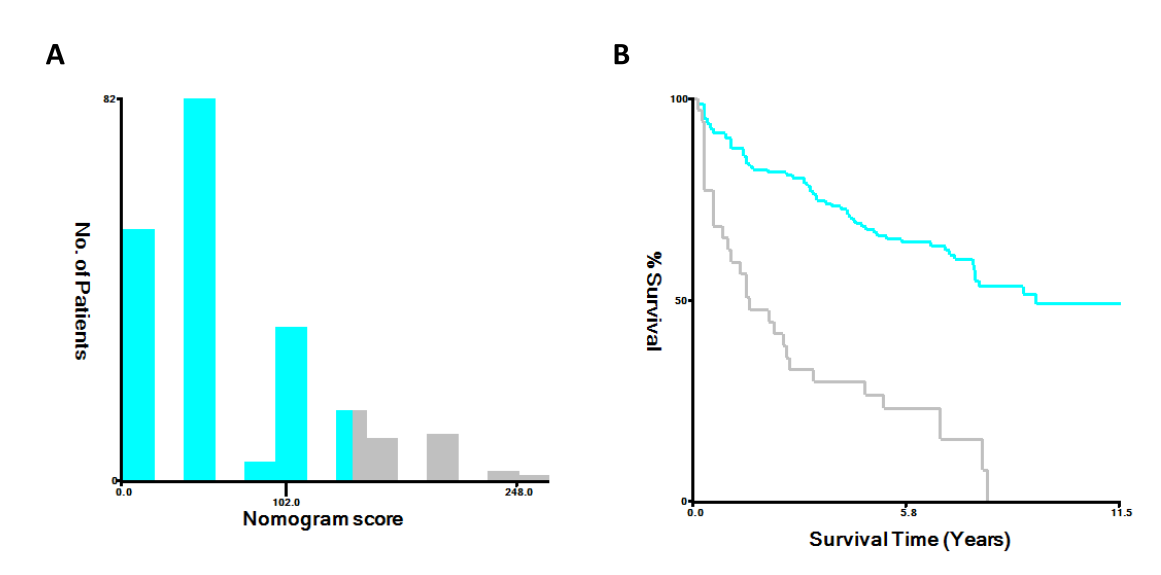


**Supplementary Figure 6. Identification of the optimal cut-off values of total scores for the nomogram using the X-tile software.** (A) The histogram shows the optimal cut-off points of the total score. (B) Kaplan-Meier curve corresponding to the cut-off points for the training cohort.

**
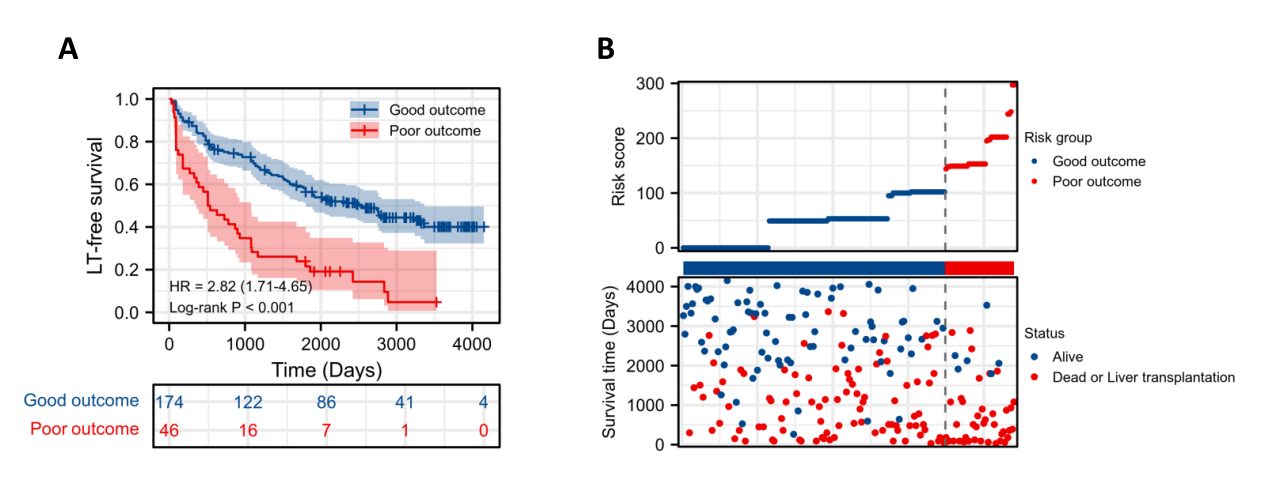
**

**Supplementary Figure 7. Survival analysis of LT-free survival in patients with complete follow-up.** (A) Kaplan-Meier curves of patients with complete follow-up. (B). The distribution of risk score calculated by the nomogram and survival status of patients. LT-free survival, Liver-transplantation free survival.


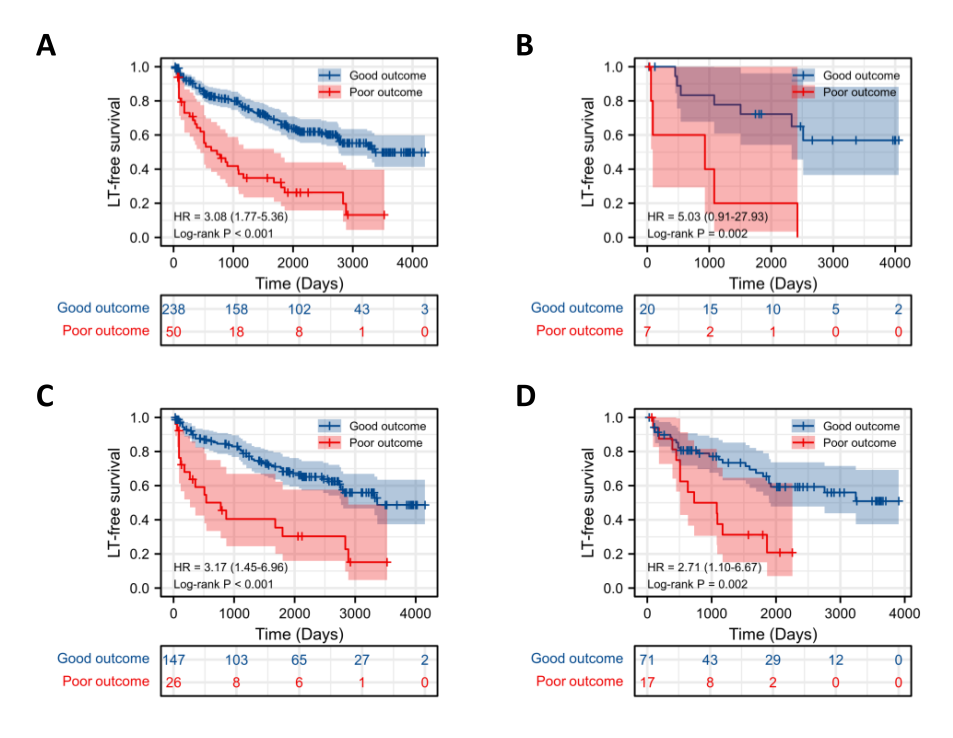


**Supplementary Figure 8. Survival analysis was performed for each subgroup in the entire cohort.** (A) HBV. (B) HCV. (C) HBV-DNA (+) and (D) HBV-DNA (-). HBV, hepatitis B virus; HCV, hepatitis C virus; LT-free survival, Liver-transplantation free survival.

.


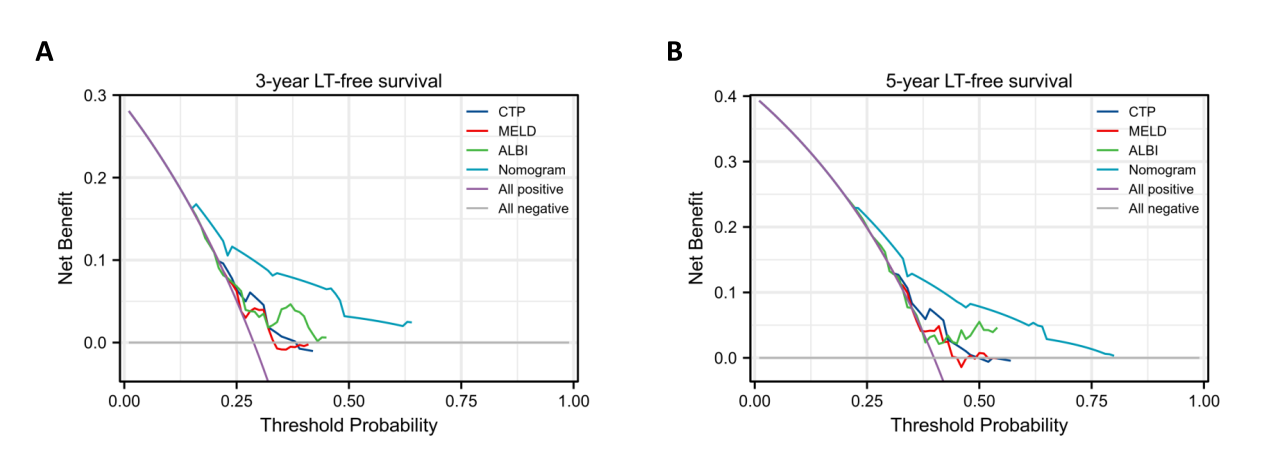


**Supplementary Figure 9. Decision curve analysis of the LT-free survival in the entire cohort.** (A) 3-year LT-free survival (B) 5-year LT-free survival. LT-free survival, Liver-transplantation free survival.


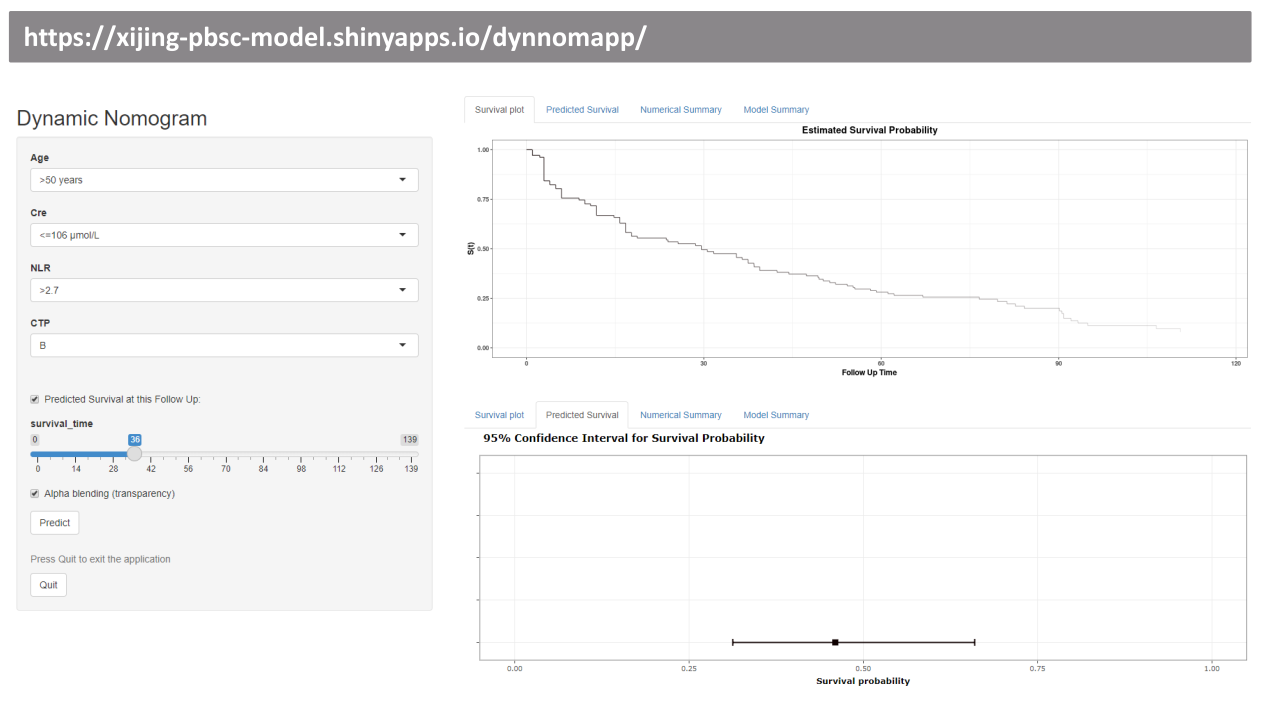


**Supplementary Figure 10. The screenshot of the web-based nomogram for the prediction of LT-free survival.** This example presents the estimated Kaplan-Meier curve and the predicted survival probability at 36 months after PBSC transplantation. The patient was older than 50 years. The patient’s Cre≤ 106 μmol/L, NLR>2.7. Besides, he or she had a liver function of CTP class B. LT-free survival, Liver-transplantation free survival; PBSC, peripheral blood stem cell; Cre, Creatinine; NLR, neutrophil-to-lymphocyte ratio; CTP, Child-Turcotte-Pugh.
